# The truth of the matter: will immune-tolerant chronic hepatitis B patients benefit from antiviral treatment?

**DOI:** 10.1097/HC9.0000000000000060

**Published:** 2023-02-14

**Authors:** Wen-Juei Jeng, Grace Lai-Hung Wong

**Affiliations:** 1 Department of Gastroenterology and Hepatology, Chang Gung Memorial Hospital, Linkou Medical Center, College of Medicine, Chang Gung University, Taoyuan City, Taiwan; 2 Medical Data Analytics Centre (MDAC), Department of Medicine and Therapeutics, and State Key Lab of Digestive Diseases, The Chinese University of Hong Kong, Hong Kong

Immune-tolerant (IT) phase is the first stage of the natural history of chronic hepatitis B (CHB) infection, also known as HBeAg seropositivity with high HBV DNA level (typically >7-8 log_10_ IU/mL) and normal ALT [below the upper limit of normal (ULN)] on more than 1 occasion over a 6–12-month period. This phase usually persists for 2–3 decades in CHB patients infected perinatally, followed by transition to the immune-active (IA) phase with increased ALT level (>2× ULN) along with decreasing HBV DNA level followed by HBeAg seroconversion. Since HBeAg seroconversion is usually followed by inactive infection and occurrence at an earlier age leads to excellent prognosis,^1^ HBeAg seroconversion is 1 of the major endpoints for HBeAg-positive patients receiving antiviral therapy. Current guidelines recommend that patients in the IT phase can be monitored and treatment initiated only if there is evidence of significant inflammation and/or fibrosis, persistence of the IT phase after the age of 30 or 40 years, or a family history of HCC.^2^–^4^ Whether all CHB patients, including those in the IT or indeterminate phase, should be treated has been hotly debated in recent years.

The argument for expanding treatment indication to IT patients is based on 4 reasons: first, REVEAL cohort reported a biological gradient of HCC development based on HBV DNA level: the higher the HBV DNA level, the higher the risk of HCC incidence.^5^ Second, inflammation and fibrosis may exist in patients with normal ALT levels. In a recent meta-analysis of 9377 CHB patients, significant fibrosis/advanced fibrosis was found in 22.3% of IT patients though none had cirrhosis.^6^ Third, HBV integration and clonal hepatocyte expansion could be seen in the IT phase, which may contribute to carcinogenesis,^7^ and antiviral treatment may decrease the integrated HBV DNA. Last, HBV-specific T cells of IT patients could still proliferate and secrete Th1 cytokine by means of in vitro expansion as in IA patients, which challenges the classic definition of immune tolerance.^8^

To date, there is no direct evidence based on randomized clinical trials that antiviral therapy improves clinically important outcomes such as mortality, end-stage liver disease, and HCC. Ex vivo HBV-specific T cell immune control has not been confirmed in IT patients.^9^,^10^ IT patients generally have favorable outcomes with no/minimal risk of cirrhosis or HCC development after 5 to 10 years of follow-up.^11^–^13^ However, several studies found that the HCC risk of untreated IT patients is not inconsequential and can be as high as that in immune-active patients.

Such conflicting findings are likely due to inadvertent misclassification of the IT phase, as some studies relied on only 1 or 2 HBV DNA or ALT assessments and may have included patients with unrecognized phase transition or immune-active CHB with fluctuating ALT levels.^14^ Another major source of confusion is the cutoff used for ULN for ALT, which may differ based on the analyzer/reagent used in the assay and the reference population.^15^,^16^ Many studies use 40 U/L as the cutoff regardless of sex, though women’s ULN for ALT is lower than that of men and studies in blood donors and living liver donors who have undergone extensive screening for liver disease showed that normal ALT is lower, around 19–25 U/L for women and 30–35 U/L for men. Other studies have not explicitly excluded patients with cirrhosis who should receive treatment if HBV DNA is detected, regardless of ALT level, or patients with moderate/advanced fibrosis and fluctuating or mildly elevated (1–2× ULN) ALT. Thus, in Mason’s study showing HBV DNA integration and clonal proliferation in IT patients, 4 had ALT ~40 U/L, 2 had stage 2 fibrosis, 1 had stage 3 fibrosis, and 1 had a histologic activity score of 4.^7^ When IT patients remain untreated, HBeAg seroconversion followed by inactive phase can occur spontaneously or when patients enter the IA phase, and treatment is offered to those who fail to achieve HBeAg seroconversion after 3–6 months of observation.^10^ Although older IT patients had similar characteristics and rates of transition to the IA phase as younger IT patients,^17^ it is important to assess liver fibrosis and necroinflammation in HBeAg-positive patients who remain in the IT phase after age 35 or 40 as prolonged periods of high-level HBV replication may increase their risk of HCC.^14^ Indeed, EASL, APASL, and AASLD guidelines recommend antiviral treatment of IT patients above the age of 30, 35, and 40 years, respectively.

Treating IT patients with nucleos(t)ide analogues and peginterferon alone or in combination leads to a very low rate of HBeAg seroconversion (0% to 5%) and HBsAg seroclearance (0% to 3%) after 1 to 4 years of treatment, and incomplete HBV DNA suppression (0% to 23%), whereas a high rate of virologic and clinical relapse occurs after stopping treatment.^9^,^18^–^20^ These observations support the lack of evidence for the benefit of treating patients in the IT phase unless novel, potent therapeutics that can achieve a high rate of functional cure of HBV are available.^21^ IT patients should be monitored to determine when they transition to the immune-active phase and when treatment should be initiated.

The meta-analysis by Lee et al^22^ argues that the risk of HCC and liver complications in patients who are truly in the IT phase and who do not have cirrhosis is low, and there is no evidence that treatment would be beneficial. This is contradictory to many Korean studies that found a high risk of HCC in untreated IT patients. The strength of this meta-analysis, which included 11,903 patients from 13 studies, is based on its stringent recruitment criteria: First, only studies with at least 2 HBV DNA and ALT tests during 6 to 12 months of observation to define the IT phase were recruited; second, CHB patients with cirrhosis were excluded even if HBV DNA and ALT met the criteria for IT phase. The patients included were in line with the classic IT phenotype with a normal ALT and a high HBV DNA level [median (range): 8.1 (6.9-9.8) log_10_ IU/mL]. The major discrepancy between this meta-analysis and Kim’s^23^ study (which included 413 patients showing the risk of HCC in untreated IT patients was higher than that of treated IA patients) is the potential mis-classication of IA patients as IT in Kim’s study, in which 26% of their IT patients had HBV DNA level < 7 log_10_ IU/mL and 25% had lower platelet count. The mean age of Kim’s IT patients was similar to that of the IA patients (38 vs. 40 y), and many exceeded the age threshold for treatment according to EASL, APASL, and AASLD guidelines. Furthermore, the subset of typical IT patients (HBV DNA >8 log_10_ IU/mL and age <30 y) had a minimal risk of HCC.^23^

This meta-analysis has certain limitations: First, studies included in this meta-analysis were retrospective in design. Second, HBeAg-positive patients with significant/advanced fibrosis but not cirrhosis might have been included since neither histologic nor noninvasive assessment of fibrosis was available in those studies. Third, the studies included some IT patients older than 30 or 40 who are recommended to undergo antiviral therapy even in the absence of significant inflammation and/or fibrosis. Fourth, the studies were heterogenous, but the authors dismissed that concern even though visual inspection of their forest plot showed that heterogeneity might impact the conclusions.

To settle this hot topic, if IT CHB patients should receive antiviral therapy, ideally, an adequately powered randomized controlled trial should be conducted, but this will require a large number (hundreds or thousands) of patients to be followed for ≥10 years, making it unethical and unfeasible. Instead, going forward, researchers on this topic should strive to use stringent criteria for the IT phase: HBeAg seropositivity, HBV DNA >7 log_10_ IU/mL, and normal ALT using sex-specific cutoff, confirmed on 1 to 2 follow-up tests over 6 to 12 months; exclude patients with cirrhosis, and stratify outcomes by age at enrollment, for example, ≤31-40 and >40 years. With the increasing global prevalence of fatty liver, histologic or noninvasive assessment of hepatic steatosis and fibrosis may be necessary to differentiate CHB patients with mildly elevated ALT due to HBV or fatty liver, as antiviral therapy alone may not benefit the latter patients. In the absence of more concrete evidence to support a benefit in treating true IT patients, current guidelines for monitoring IT patients remain valid until novel therapies with a high rate of functional cure become available.
